# Endogenous zebrafish proneural Cre drivers generated by CRISPR/Cas9 short homology directed targeted integration

**DOI:** 10.1038/s41598-021-81239-y

**Published:** 2021-01-18

**Authors:** Maira P. Almeida, Jordan M. Welker, Sahiba Siddiqui, Jon Luiken, Stephen C. Ekker, Karl J. Clark, Jeffrey J. Essner, Maura McGrail

**Affiliations:** 1 Department of Genetics, Development and Cell Biology, Iowa State University, Ames, IA USA; 2 Genetics and Genomics Interdepartmental Graduate Program, Iowa State University, Ames, IA USA; 3 Department of Biochemistry and Molecular Biology, Mayo Clinic, Rochester, MN USA; 4 Present Address: Department III – Developmental Genetics, Max Planck Institute for Heart and Lung Research, Bad Nauheim, Germany

**Keywords:** Genetics, Functional genomics

## Abstract

We previously reported efficient precision targeted integration of reporter DNA in zebrafish and human cells using CRISPR/Cas9 and short regions of homology. Here, we apply this strategy to isolate zebrafish Cre recombinase drivers whose spatial and temporal restricted expression mimics endogenous genes. A 2A-Cre recombinase transgene with 48 bp homology arms was targeted into proneural genes *ascl1b*, *olig2* and *neurod1*. We observed high rates of germline transmission ranging from 10 to 100% (2/20 *olig2*; 1/5 *neurod1*; 3/3 *ascl1b*). The transgenic lines *Tg*(*ascl1b-2A-Cre)*^*is75*^, *Tg*(*olig2-2A-Cre)*^*is76*^, and *Tg*(*neurod1-2A-Cre)*^*is77*^ expressed functional Cre recombinase in the expected proneural cell populations. Somatic targeting of 2A-CreERT2 into *neurod1* resulted in tamoxifen responsive recombination in the nervous system. The results demonstrate Cre recombinase expression is driven by the native promoter and regulatory elements of the targeted genes. This approach provides a straightforward, efficient, and cost-effective method to generate cell type specific zebrafish Cre and CreERT2 drivers, overcoming challenges associated with promoter-BAC and transposon mediated transgenics.

## Introduction

The Cre/*lox* recombinase system has been widely used in zebrafish for spatiotemporal control of gene expression and lineage tracing^1–4^. The effectiveness of the system is dependent on drivers that provide precise spatial and temporal Cre expression, and recent technical advances combining multiple recombinases have increased the ability to restrict activity to defined cell populations^5,6^. However, the approach is still limited by methods used to isolate zebrafish transgenics which are susceptible to position effect and multigenerational silencing. An improved method to isolate recombinase transgenics that recapitulate endogenous gene expression patterns would increase the robustness and reproducibility of Cre/lox lineage tracing and gene function studies.

In organisms such as zebrafish that lack embryonic stem cell methodology, Tol2-mediated transgenesis^7,8^ has traditionally been used to isolate Cre drivers^9^. In one approach, a gene promoter is cloned or BAC-engineered into a promoter-Cre fusion inside a Tol2 vector^4,10^. Alternative strategies using Tol2 transgenesis exploit the nearly unbiased, genome wide random integration of the transposon to isolate novel cell type and tissue-specific drivers. Enhancer trap Tol2 vectors contain a minimal promoter-Cre-2A-reporter cassette and are designed to drive expression under the control of local enhancer elements at the integration site^11,12^. Tol2 vectors engineered with a splice acceptor-mCherry-2A-Cre-ERT2 gene trap are driven by endogenous promoter and regulatory elements after in-frame integration within a gene^13^. Although each Tol2-based method allows for efficient recovery of transgenics, difficulties associated with defining and cloning a complete promoter and associated regulatory elements, combined with multicopy transgene integration, position effects and gene silencing, limits the effectiveness of creating drivers with consistent restricted expression patterns^3,14,15^.

Recent advances in gene editing with engineered site-specific nucleases may solve a number of the limitations associated with Tol2 transgenic approaches used to isolate zebrafish transgenic Cre drivers. The ability of TALENs and CRISPR/Cas9 to direct a double stand break to a specific location in the genome and generate single stranded overhangs for DNA repair has been exploited to introduce exogenous DNA at the target site via non-homologous end-joining (NHEJ) and homology directed repair (HDR) pathways^16–20^. Early studies in zebrafish demonstrated the addition of complementary homology arms flanking a donor transgene significantly increased the frequency and precision of targeted integration^21–23^. One report in zebrafish described using CRISPR and 1 kb of homology cloned in front of a targeting cassette to integrate Cre-ERT2 upstream of the *otx2* translation initiation codon^24^. However, the efficiency of precision integration at multiple loci using this approach is not known.

To streamline isolation of zebrafish Cre drivers for specific cell lineages and cell types, we applied our recently reported method for CRISPR/Cas9 short homology directed targeted integration^25^ to generate Cre transgenic lines. As proof of principle we targeted a 2A-Cre cassette in frame into a coding exon in the proneural transcription factor genes *ascl1*, *olig2* and *neurod1* that define neural stem, progenitor and post-mitotic cell lineages^26–32^. We chose to target *ascl1b* and *olig2* since both are differentially expressed in highly aggressive pediatric brain cancers characterized by embryonal-like, poorly differentiated tumors^33,34^, consistent with their role in progenitor cell function during neural development. We and others had shown previously *ascl1b* and *olig2* are overexpressed in zebrafish embryonal brain tumor models, reinforcing the conservation of molecular mechanisms driving neuroectodermal tumor types^35,36^. Together with *neurod1*, a marker of early neural commitment and differentiation, *ascl1b* and *olig2* are good candidates to generate Cre drivers that would be useful for functional studies in neurogenesis and brain tumor pathogenesis. The 2A-Cre and 2A-CreERT2 targeting vectors used for integration are part of our pPRISM vector series that contains cassettes with linked fluorescent secondary marker for allele tracking (Welker et al., in preparation). Recovery of 2A-Cre integration alleles was highly efficient, with frequencies ranging from 10 to 100%, similar to other cargos as reported previously^25^. As expected, the expression of functional Cre recombinase was restricted to cell populations defined by *ascl1b*, *olig2* and *neurod1*. Somatic targeting of Cre-ERT2 into *neurod1* shows tamoxifen inducible recombinase activity in the nervous system. Together, our results demonstrate CRISPR/Cas9 directed targeted integration is an efficient and robust method for isolating endogenous Cre drivers that reflect the endogenous promoter activity of the targeted genes.

## Results and discussion

### CRISPR/Cas9 mediated Knock-in of 2A-Cre into *ascl1b*, *olig2*, and *neurod1*

**Figure 1:**
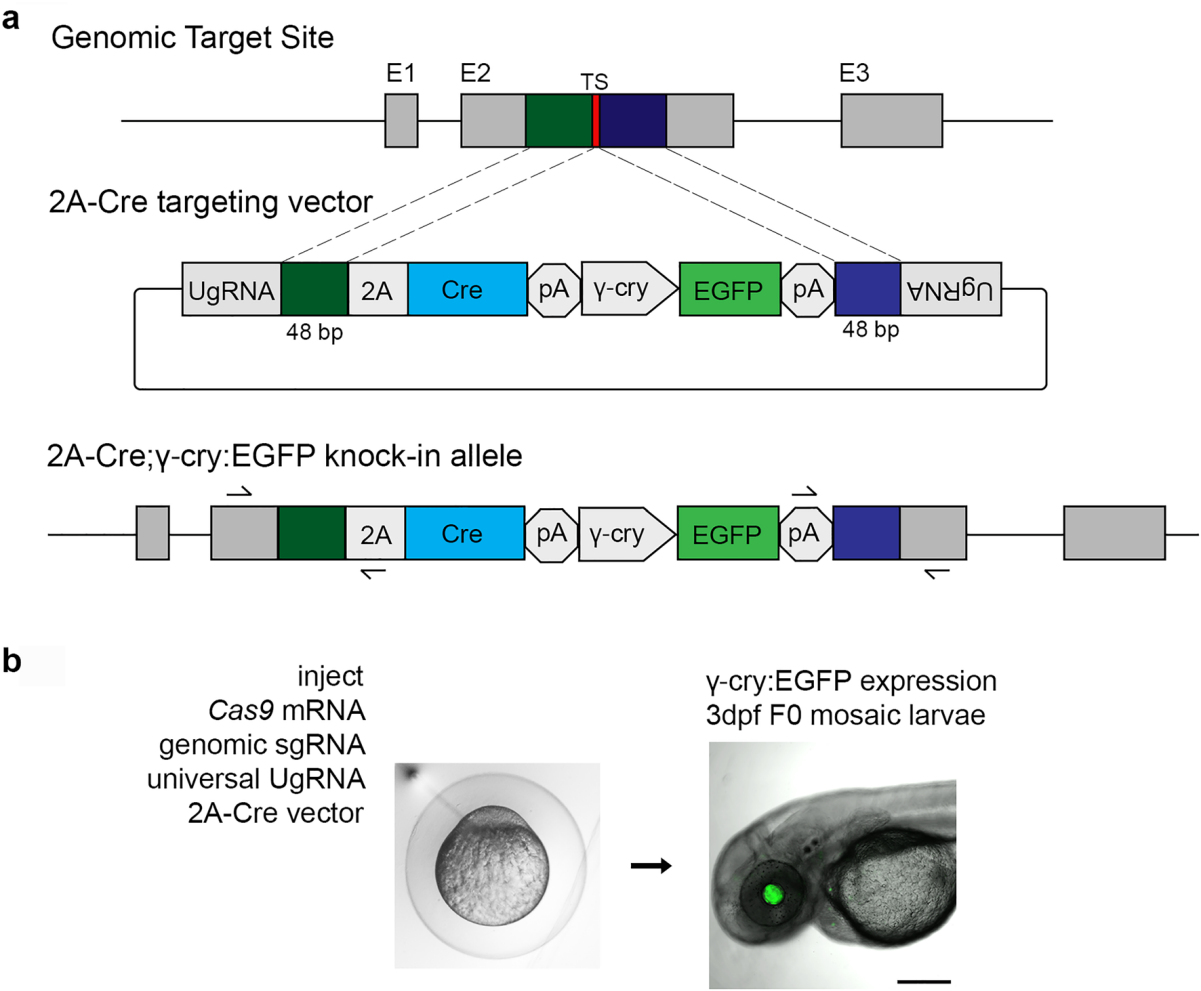
CRISPR/Cas9 short homology directed targeted integration strategy for efficient recovery of Cre knock-in alleles. (**a**) Schematic of the genomic target site, Cre donor vector, and final knock in allele. A CRISPR sgRNA (red) was chosen in the coding sequence. 48 bp upstream (dark green) and 48 bp downstream (dark blue) of the cut site are included in the donor vector as homology arms. The donor vector has UgRNA sequences flanking the homology arms for in vivo liberation of the knock in cassette. The knock-in cassette contains an in frame 2A-Cre for expression of Cre recombinase, and a secondary marker driving *γ-cry:EGFP* expression in the lens. Arrows indicate PCR primers for 5′ and 3′ genome/vector junction analysis. (**b**) 1 cell stage zebrafish embryos are injected with Cas9 mRNA (300 pg), genomic sgRNA (25 pg), UgRNA (25 pg), and 2A-Cre targeting vector (10 pg). At 3 days post fertilization, embryos expressing EGFP in the lens are selected and raised to adulthood. *TS* genomic sgRNA target site, *UgRNA* universal short guide RNA target site, γ-*cry* gamma crystallin promoter, *EGFP* green fluorescent protein, *pA* transcription termination and polyadenylation sequence.

Proneural Cre drivers were generated by integration of a 2A-Cre recombinase cDNA cassette in frame into a coding exon of the zebrafish *ascl1b*, *olig2* and *neurod1* genes (Fig. 1). We used our recently published strategy for CRISPR/Cas9 precision targeted integration with short homology arms that is likely to drive integration by homology mediated end joining (HMEJ)^25^. The 2A-Cre targeting construct contains a secondary fluorescent marker cassette with the *γ-crystallin* (*γ-cry*) promoter driving EGFP expression (Fig. 1a). Efficient targeted integration after injection into embryos is driven by in vivo liberation of the homology arms flanking the cassette, which are released by Cas9 induced double strand breaks at the universal gRNA (UgRNA) sites in the vector (Fig. 1a).

sgRNAs were designed to CRISPR/Cas9 sites in exon 1 of *ascl1b* and exon 2 of *olig2* and *neurod1* (Table 1). The sgRNA mutagenesis efficiency was tested by co-injection with Cas9 mRNA into 1-cell zebrafish embryos, followed by PCR amplification of the targeted exon and analysis of heteroduplex formation in the PCR product by gel electrophoresis. After confirmation of efficient mutagenesis, 48 bp of sequence on either side of the genomic target Cas9 was used to design and clone 5′ and 3′ homology arms into the 2A-Cre targeting vector. Zebrafish embryos were injected at the 1-cell stage with the genomic target site sgRNA, the UgRNA, Cas9 mRNA, and the 48 bp homology arm 2A-Cre targeting vector. At 3 days post-fertilization (dpf), larvae were screened for expression of the lens specific γcry:EGFP secondary marker (Fig. 1b), and positive larvae selected to raise to adulthood to test for germline transmission. Targeting of *ascl1b*, *olig2* and *neurod1* resulted in 58%, 48% and 37%, respectively, of F0 injected embryos showing EGFP expression in the lens (Table 1), demonstrating the presence of the injected targeting vector. Evidence of somatic targeted integration at the genomic site was confirmed by junction fragment PCR on injected embryo.

**Table 1:** Efficient recovery of endogenous neurogenic Cre driver lines by 2A-Cre-γcry:EGFP targeted integration.

Neurogenic gene	*ascl1b*	*olig2*	*neurod1*
Targeted exon	1	2	2
5′ and 3′ homology arms (bp/bp)	48/48	48/48	48/48
F0 embryos expressing secondary marker	58% (61/105)	48% (52/109)	37% (21/57)
F0 adults transmitting secondary marker	100% (3/3)	10% (2/20)	20% (1/5)
F0 adults transmitting precise integration allele	33% (1/3)	5% (1/20)	20% (1/5)

To test whether somatic integration of the 2A-Cre cassette led to expression of functional Cre recombinase in the expected neural cell populations, we injected the genomic sgRNA, UgRNA, Cas9 mRNA, and the donor vector into embryos from the transgenic ubi:Switch floxed reporter line *Tg(ubi:loxP-EGFP-loxP-mCherry)* (Mosimann et al*.*, 2011). Injected F0 mosaic *Tg(ubi:loxP-EGFP-loxP-mCherry*) embryos showed a switch from EGFP to mCherry expression in cells throughout the brain and retina, while EGFP expression remained in cells outside of the central nervous system (Supplementary Fig. S1). These results suggested that Cre expression was controlled by the endogenous regulatory elements of the targeted proneural genes, leading to recombination in the specific neural progenitor populations defined by those genes. Furthermore, the results suggest that the expressed Cre has recombinase activity that leads to recombination at *loxP* sites and excision of the floxed *EGFP* cassette. All neural progeny descended from the neural progenitor populations inherit the recombination event and express mCherry. The observed recombination in F0 injected animals indicated on-target integration of the 2A-Cre cassette was relatively efficient. Together, these results suggested targeted integration would be an effective method to generate endogenous Cre drivers that can promote spatially restricted cell-type specific Cre-mediated recombination.

Stable germline 2A-Cre knock-in alleles were established from adult F0s outcrossed to wildtype WIK to identify individuals transmitting the γcry:EGFP secondary marker to their progeny. We first measured the frequency of germline transmission of the γcry:EGFP secondary marker and found rates for *ascl1b*, *olig2* and *neurod1* of 100%, 10% and 20%, respectively (Table 1). F1 embryos positive for EGFP expression were tested for on-target precise integration at the genomic target site by PCR amplification and sequencing of the 5′ and 3′ genome/cassette junctions (Fig. 1, Supplementary Fig. S2). We found F0 transmission of precise integration alleles through the germline was relatively high, with frequencies at *ascl1b*, *olig2* and *neurod1* of 33% (1/3), 5% (1/20) and 20% (1/5), respectively (Table 1). These results at 3 independent loci demonstrate 2A-Cre in-frame targeted integration alleles can be efficiently recovered after screening a minimum of 20 F0 adults.

Single F1 adults harboring precise integration alleles were outcrossed to recover F2 adults, and single F2 adults were used to establish *Tg*(*ascl1b-2A-Cre)*^*is75*^, *Tg*(*olig2-2A-Cre)*^*is76*^, and *Tg*(*neurod1-2A-Cre)*^*is77*^ F3 families. Because the 2A-Cre cassette was integrated in the proneural gene coding sequence, a loss of function mutation should result; therefore, the transgenic lines are maintained as heterozygotes. The F3 heterozygous *ascl1b-2A-Cre*, *olig2-2A-Cre*, and *neurod1-2A-Cre* larvae and adults did not exhibit anatomical or behavioral phenotypes.

### Endogenous proneural 2A-Cre transgenic lines express Cre in a pattern that recapitulates the targeted gene

**Figure 2:**
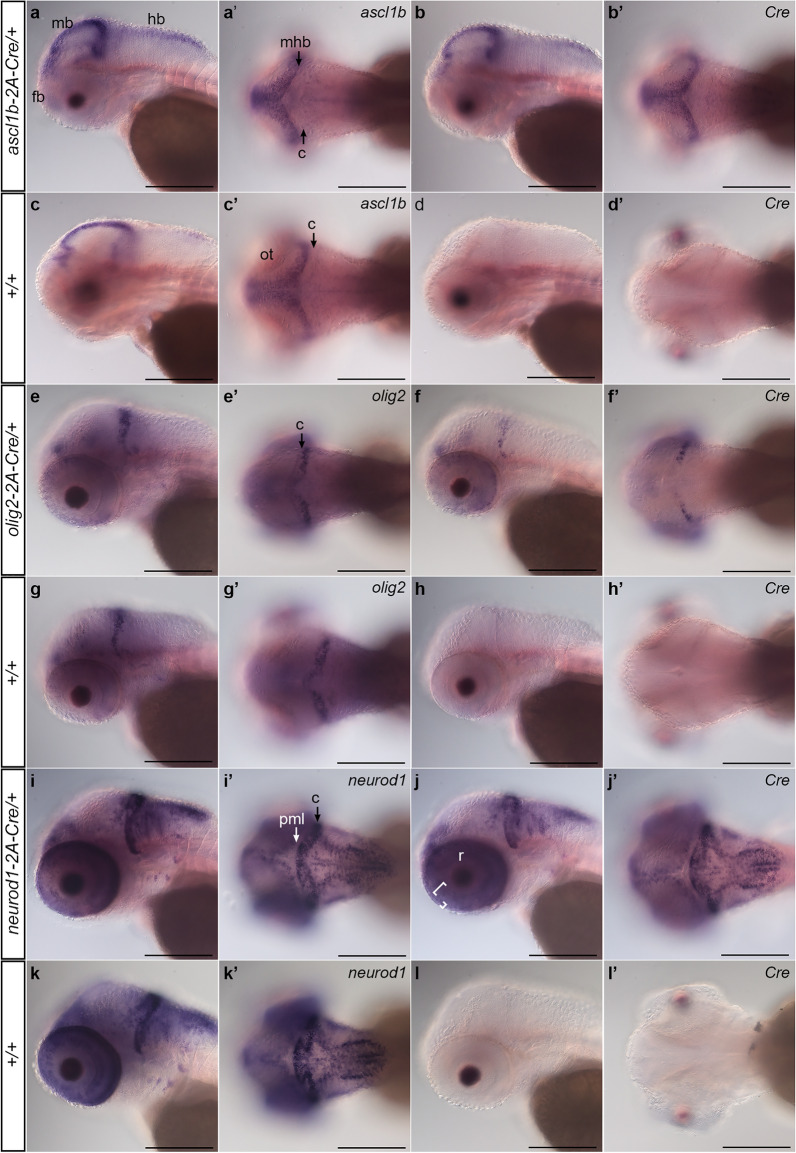
Expression of 2A-Cre integration alleles recapitulates *ascl1b*, *olig2*, and *neurod1* expression pattern. Whole mount in situ hybridization for endogenous genes and Cre was performed in 3 dpf larvae obtained from outcrossing *ascl1b-2A-Cre/*+, *olig2-2A-Cre/*+, and *neurod1-2A-Cre/*+ lines to wild type WIK. Sibling larvae from the same clutch were sorted into lens EGFP-positive and -negative groups before fixation. For each genotype and probe 3 individual larvae were photographed. (**a**–**d**) *ascl1b* and Cre expression in *ascl1b-2A-Cre/*+ *larvae* show similar patterns in the forebrain, and along the midbrain ventricle and midbrain-hindbrain border. (**e**–**g**) *olig2* and Cre expression in *olig2-2A-Cre/*+ larvae were restricted to the forebrain, the posterior half of the cerebellum, and a subset of cells in the retina. (**i**–**k**) *neurod1* and Cre expression in *neurod1-2A-Cre/*+ larvae were detected in the forebrain, adjacent to the midbrain and hindbrain ventricles and enriched in the cerebellum ((**i**′), C black arrow), and in the retina inner (large bracket) and outer (small bracket) nuclear layers (**j**). *neurod1* and Cre expression were not detected in the posterior peripheral midbrain layer ((**i**′), PML white arrow). Cre expression was not detected in wild type +/+ sibling larvae (**d**,**h**,**l**). *c* cerebellum, *fb* forebrain, *hb* hindbrain, *mb* midbrain, *mhb* midbrain-hindbrain border, *nt* neural tube, *ot* optic tectum, *pml* peripheral midbrain layer, *r* retina. Scale bar 250 μm.

To confirm the *ascl1b-2A-Cre*, *olig2-2A-Cre*, and *neurod1-2A-Cre* integration lines express Cre in a pattern that recapitulates the targeted gene, we used whole mount in situ hybridization to examine *Cre* transcript localization. Adult F2 or F3 heterozygotes were outcrossed to wild type WIK and 3 dpf sibling larvae were hybridized with a probe complementary to *Cre* or to the target gene mRNA. Each of the lines showed that the pattern of Cre expression was identical to the endogenous gene mRNA (Fig. 2).

*ascl1b-2A-Cre/*+ larvae hybridized with *ascl1b* or *Cre* probes showed the same pattern of signal in the forebrain, along the midbrain ventricle and midbrain-hindbrain border in the optic tectum, and on the dorsal surface of the hindbrain (Fig. 2a–d), as previously reported^37^. *olig2-2A-Cre*/+ larvae showed *Cre* expression matched *olig2* in wild type larvae (Fig. 2e–h)^38^, and was restricted to the posterior half of the cerebellum, a subset of cells in the neural retina, and the ventral forebrain. Like the endogenous *neurod1* in wild type (Fig. 2i,j)^39,40^, *Cr*e expression in *neurod1-2A-Cre/*+ was observed in the forebrain, adjacent to the midline in the midbrain and hindbrain, and absent from the peripheral midbrain layer of the optic tectum, consistent with *neuro*d1 expression in committed progenitors and newborn post-mitotic neurons. It was highly expressed in the cerebellum and in the inner and outer neural layers of the retina (Fig. 2i–l). For all three lines, the consistency between the *Cre* and endogenous gene expression patterns suggested that both were controlled by the same regulatory elements, providing gene specific spatial and temporal Cre expression in neural progenitor populations defined by the proneural transcription factors.

### Endogenous neurogenic 2A-Cre transgenic lines lead to efficient Cre-*loxP* recombination in the expected neural cell populations defined by proneural gene expression

**Figure 3:**
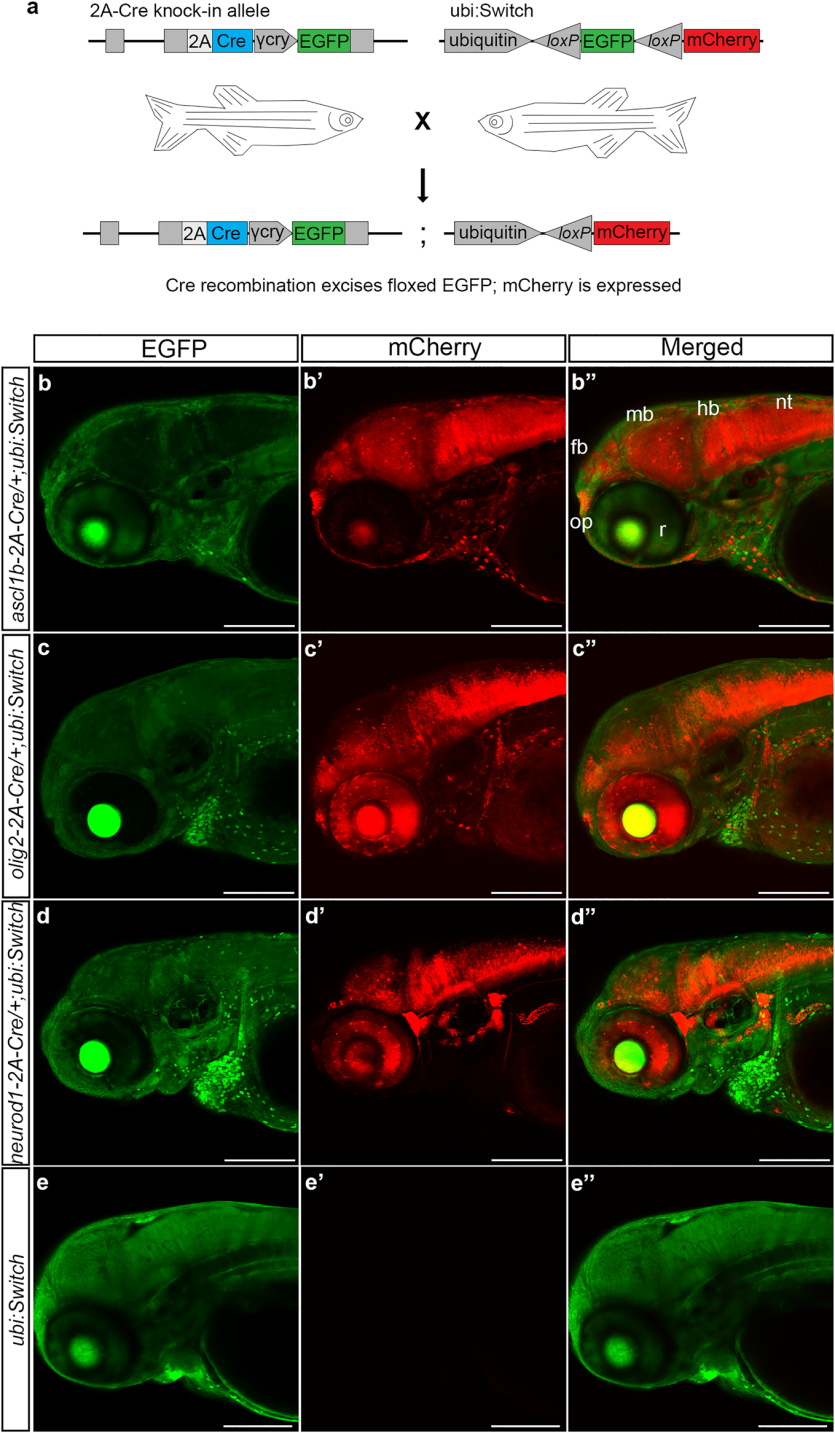
Transgenic *ascl1b-2A-Cre*, *olig2-2A-Cre* and *neurod1-2A-Cre* lines express functional Cre that promotes recombination at *loxP* sites in the expected neural progenitor populations. (**a**) F0 adults harboring precise integration alleles were mated to the recombination reporter line ubi:Switch to generate double transgenic 2A-Cre driver; ubi:Switch embryos. (**b**–**d**) Confocal imaging of 3 dpf double transgenic *ascl1b-2A-Cre*; *ubi:switch* (**b**), *olig2b-2A-Cre*; *ubi:switch* (**c**), and *neurod1-2A-Cre*; *ubi:switch* (**d**) larvae shows a switch from GFP to mCherry expression in neural cells derived from *ascl1b*, *olig2*, and *neurod1* progenitors. (**e**) A switch from GFP to mCherry expression isn’t detected in single transgenic *ubi:Switch* larvae. *fb* forebrain, γ*-cry* gamma crystallin promoter, *EGFP* green fluorescent protein, *hb* hindbrain, *mb* midbrain, *nt* neural tube, *op* olfactory placode, *r* retina. Scale bar 200 μm.

To confirm the stable *ascl1b-2A-Cre*, *olig2-2A-Cre*, and *neurod1-2A-Cre* driver lines express functional Cre recombinase in the expected neural progenitor cell populations, F0 adults were mated to the ubi:Switch line (Fig. 3a). The offspring were imaged at 3 dpf and showed expression had switched from EGFP to mCherry in neurons in the developing forebrain, midbrain, hindbrain and retinas of the double heterozygous 2A-Cre; ubi:switch larvae (Fig. 3b–d). *ascl1b-2A-Cre* led to near complete switching of EGFP to mCherry along the anterior–posterior axis of CNS, from the olfactory placode extending to the neural tube, with the exception of the neural retina (Fig. 3b). This is consistent with the early expression of *ascl1b* in the midbrain and hindbrain regions of the neural plate in 4–10 somite stage embryos, which broadens to cells in the anterior telencephalon, diencephalon, midbrain tegmentum and hindbrain by 25 somites/22 h post fertilization, but is completely absent from the developing retina^41,42^. Little switching occurred in cells outside of the CNS. These results indicated Cre expressed by the *ascl1b-2A-Cre* allele led to efficient recombination at the *ubi:Switch* transgene *loxP* sites and excision of the floxed EGFP cassette.

Similar to *ascl1b-2A-Cre*, the *olig2-2A-Cre* transgenic line led to recombination and switching from EGFP to mCherry expression throughout the CNS, including the retina (Fig. 3c). The pattern was consistent with early expression of *olig2* in the neural plate presumptive ventral diencephalon at 8 hpf^43^; forebrain dorsal thalamus, subpallium, posterior ventral thalamus and posterior tuberculum at 24–30 hpf^44^; and along the forebrain ventricles, retina, midbrain/hindbrain border and cerebellum at 48 hpf^45,46^. The pattern of switching induced by the *neurod1-2A-Cre* line extended throughout neurons in the forebrain, midbrain, hindbrain, retina, and the PNS (Fig. 3d), as expected, given *neurod1* is expressed in committed neural progenitors and early post mitotic neurons throughout the nervous system^47^. In single transgenic ubi:Switch larvae switching does not occur and only EGFP expression is detected (Fig. 3e).

**Figure 4:**
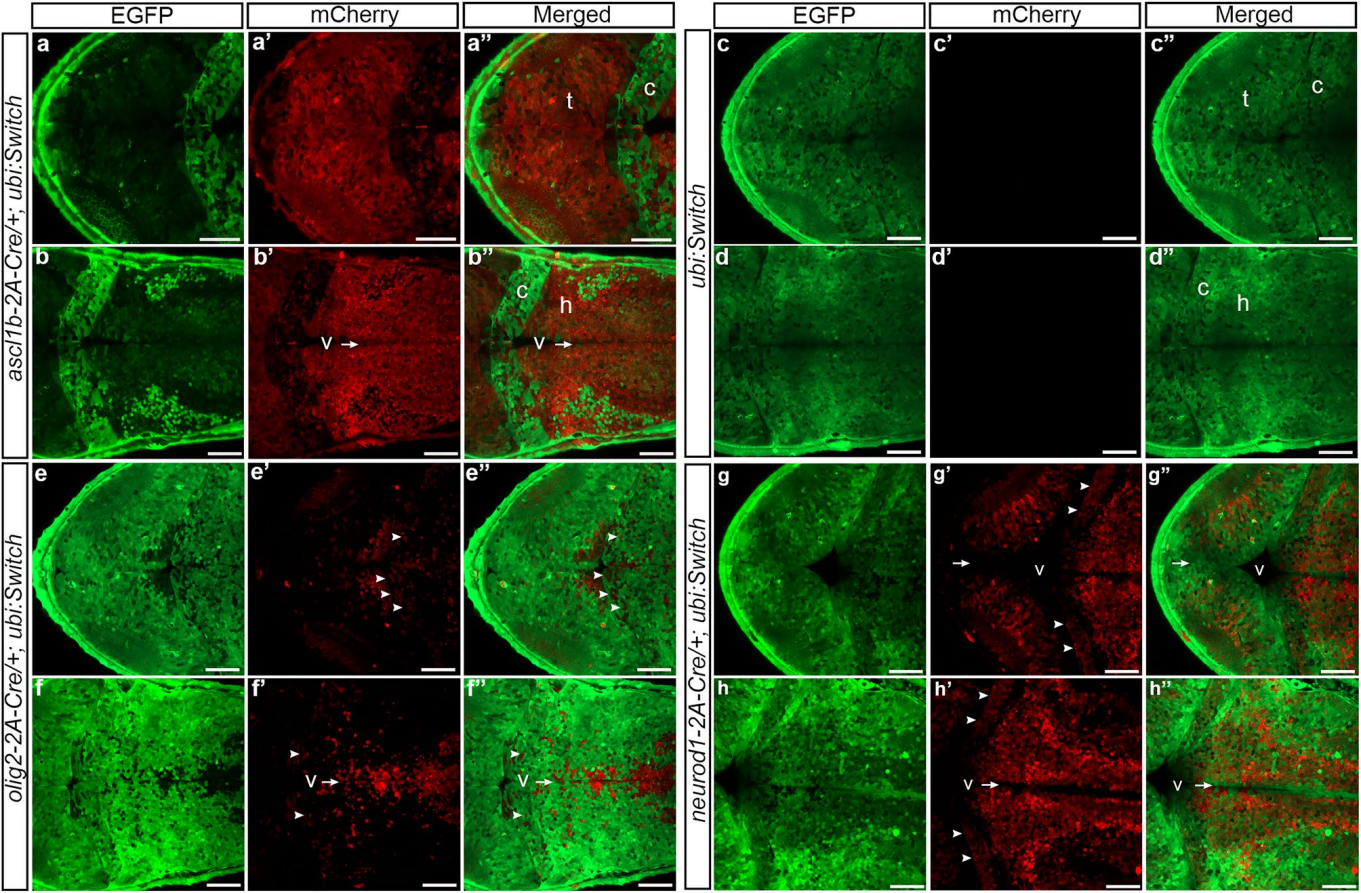
*ascl1b-2A-Cre*, *olig2-2A-Cre* and *neurod1-2A-Cre* Cre recombinase activity in the 3 dpf larval midbrain and hindbrain. F3 or F2 adults were mated to the recombination reporter line ubi:Switch to generate double transgenic embryos. Confocal imaging of *ascl1b-2A-Cre; ubi:Switch* (**a**–**a**′′,**b**–**b**′′), control sibling *ubi:Switch* (**c**,**d**), *olig2-2A-Cre; ubi:Switch* (**e**–**e**′′,**f**–**f**′′) and *neurod1-2A-Cre; ubi:Switch* (**g**–**g**′′,**h**–**h**′′) larval midbrain tectum (t), cerebellum (c) and hindbrain (h). Arrows point to the ventricular zone lining the midbrain and hindbrain ventricles (v). Expression of *ascl1b-2A-Cre* earlier in brain development leads to nearly all descendant neurons expressing mCherry (**a**,**b**). No switching occurs in the absence of Cre (**c**–**c**′′,**d**–**d**′′). *olig2-2A-Cre* leads to mCherry expression in a subset of cells in the cerebellum (arrowheads), and neural progenitors lining the hindbrain ventricular zone and their descendants (**e**,**f**). In *neurod1-2A-Cre* mCherry expression is absent from the ventricular zone (v arrows) but present throughout neurons in the tectum, cerebellum (arrowheads) and hindbrain (**g**,**h**). Scale bars 50 μm.

Higher resolution analyses of recombinase switching in the *ascl1b-2A-Cre*, *olig2-2A-Cre*, and *neurod1-2A-Cre* 3 dpf larval midbrain and hindbrain were consistent with cell-specific expression of Cre recombinase (Fig. 4). F3 *ascl1b-2A-Cre*, F2 *olig2-2A-Cre*, and F2 *neurod1-2A-Cre* adults were crossed to the *ubi:Switch* line and RFP expression was examined in the 3 dpf larval dorsal tectum, cerebellum and hindbrain. In *ascl1b-2A-Cre; ubi:Switch* nearly all cells throughout the tectum (Fig. 4a–a′′), including cells lining the ventricle, expressed mCherry, consistent with the high levels of *ascl1b* and *Cre* transcripts detected in the ventricular zone of the midbrain by in situ hybridization (Fig. 3a–d). A subset of cells in the cerebellum had switched from EGFP to mCherry expression (Fig. 4a′′,b′′), which may be from the small number of cells expressing *ascl1b* in the cerebellum (Fig. 3a′,b′,c′), or the result of earlier recombination in progenitors during embryonic brain development. mCherry expression was detected throughout the hindbrain, as expected given the earlier widespread expression of *ascl1b* in stem/progenitors in the neural plate and embryonic brain regions, and the expression of *ascl1b* and *Cre* transcripts detected by in situ hybridization in a subset of cells lining the ventricles and located laterally in the dorsal 3 dpf hindbrain (Fig. 3a–d). A switch from EGFP to mCherry expression wasn’t detected in sibling single transgenic *ubi:Switch* larvae (Fig. 4c–c′′,d–d′′). In *olig2-2A-Cre; ubi:Switch* the switch from EGFP to mCherry was detected in a subset of cells at the posterior region of the tectum (Fig. 4e–e′′), in the cerebellum (Fig. 4e,f arrowheads), in cells lining the hindbrain ventricle (Fig. 4f–f′′ V arrow), and in cells scattered throughout the hindbrain (Fig. 4e–e′′,f–f′′). In contrast, in *neurod1-2A-Cre; ubi:Switch* larval brains mCherry wasn’t detected in cells lining the ventricle of the tectum or hindbrain (Fig. 4g,h, arrows). mCherry was present in cells throughout the cerebellum (Fig. 4g′,h′ arrowheads) and the midbrain and hindbrain parenchyma, as expected given *neurod1* expression is induced after committed progenitors migrate away from the ventricle and transition to the neuronal state (Fig. 4g–g′′,h–h′′). The mCherry expression was consistent with the strong pattern of in situ hybridization signal for *neurod1* and *Cre* in the 3dpf midbrain and hindbrain (Fig. 3i–l). Together, these results show the 2A-Cre knock-in lines express functional Cre recombinase in the pattern and cell type defined by the targeted proneural gene, leading to progenitor cell descendants inheriting the recombined floxed allele.

**Figure 5:**
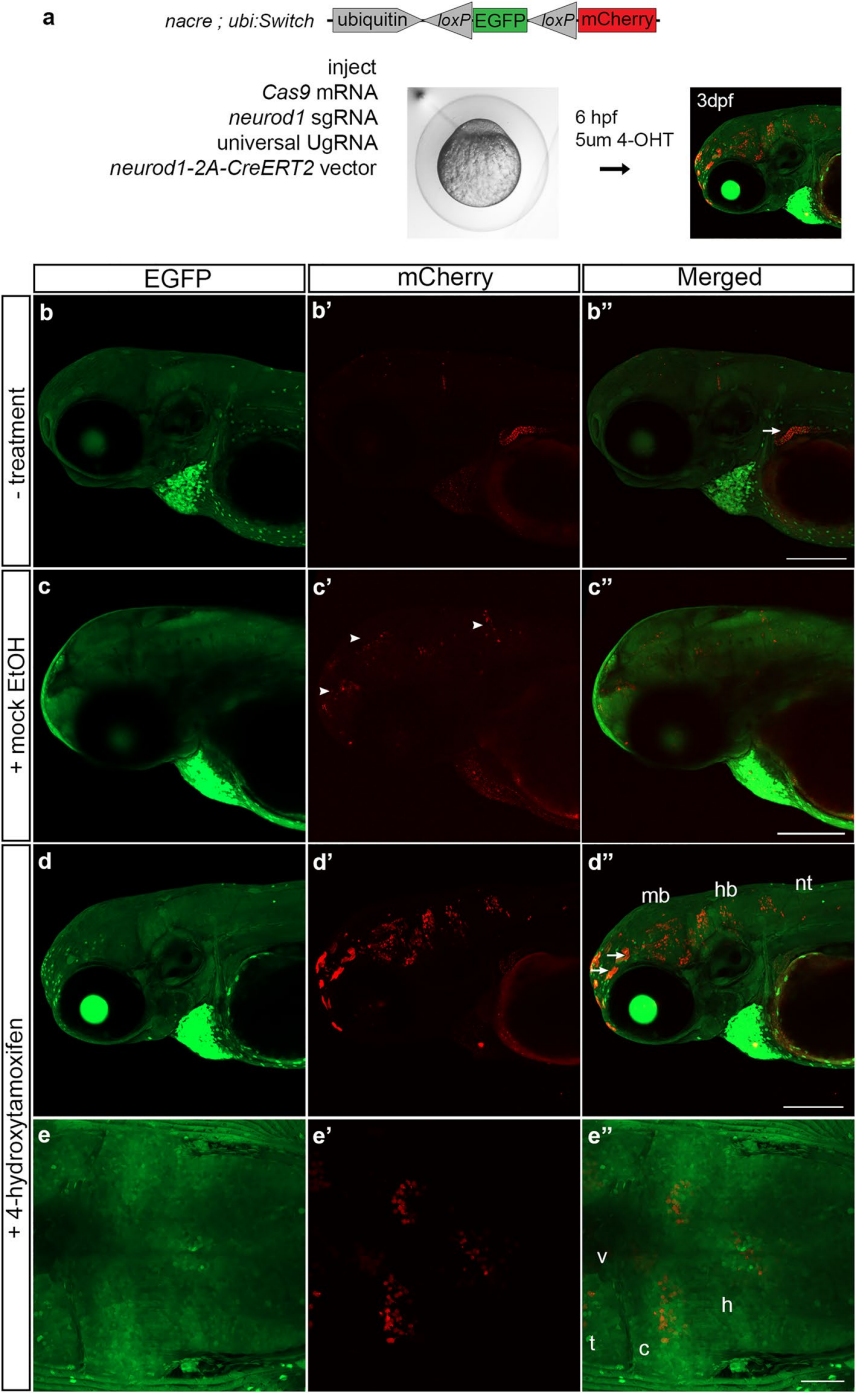
Tamoxifen regulated CreERT2 recombinase activity in F0 somatic targeted embryos. (**a**) *nacre; ubi:Switch* embryos injected with Cas9mRNA, sgRNAs and the neurod1-2A-CreERT2 targeting vector. At 6 hpf embryos were mock treated or treated with 5um 4-hydroxytamoxifen and imaged at 3 dpf. (**b**–**b**′′) Injected embryos without treatment or (**c**–**c**′′) mock treated show a low level of expression in the brain. (**d**–**d**′′) 4-hydroxytamoxifen treatment resulted in switching from EGFP to mCherry expression in cells in the midbrain, hindbrain and neural tube. Arrows point to off-target expression. (**e**–**e**′′) Dorsal imaging of the brain in the larvae shown in **d**–**d**′′ revealed mCherry expression was detected in cells throughout the midbrain and hindbrain parenchymal tissue. *4-OHT* 4-hydroxytamoxifen, *c* cerebellum, *EGFP* enhanced green fluorescent protein, *hb* hindbrain, *mb* midbrain, *nt* neural tube, *t* optic tectum, *v* ventricle. Scale bars (**b**′′,**c**′′,**d**′′) 200 μm; (**e**′′) 50 μm.

We next replaced the Cre cDNA in the 2A-Cre-PRISM vector with CreERT2, which was used previously by Mosimann et al., 2011 to generate the ubiquitin:CreERT2 transgenic by Tol2 mediated transgenesis^4^. To test whether somatic integration of CreERT2 would lead to expression of tamoxifen responsive Cre recombinase, the 2A-CreERT2 cassette was targeted to the *neurod1* exon 2 sgRNA site in *nacre*; *ubi:Switch* embryos that lack melanin pigment (Fig. 5a). At 6hpf injected embryos were treated with 5 µm 4-hydroxytamoxifen (4-OHT) in ethanol or ethanol alone and placed in a light-blocking container. On day 2 and day 3 the embryos were placed in fresh solution. At 3dpf, mCherry expression was detected in 17% (4/23) of untreated injected embryos (Fig. 5b–b′′) and 11% (2/19) of mock treated injected embryos (Fig. 5c–c′′) (Table 2), however the amount of switching was reduced compared to treated embryos. Recombination in the absence of treatment (Fig. 5b–b′′) or tamoxifen (Fig. 5c–c′′) may be caused by a stress response from injection, that alters hsp70 ability to tether CreERT2 at the cell membrane and in the cytoplasm. 45% (10/22) of injected embryos treated with 4-hydroxytamoxifen showed widespread mosaic mCherry expression throughout the brain and spinal cord (Fig. 5d–d′′,e–e′′; Table 2). Some examples of off targeting were detected in the gut (Fig. 5b′′ arrowhead) and skin (Fig. 5d′′ arrows). As expected, uninjected control embryos did not show mCherry expression (0/24) (Table 2). *neurod1-2A-CreERT2*-genomic DNA junction fragment analysis showed evidence of integration that correlates with switching in targeted embryos (Supplementary Fig. S3). Together, these results show targeted integration of Cre-ERT2 leads to endogenous expression of tamoxifen inducible Cre recombinase.

**Table 2:** Tamoxifen responsive Cre recombinase activity in neurod1-2A-CreERT2 targeted embryos.

Condition	# viable 3dpf larvae	# mcherry + 3dpf larvae
Injected	23/30	4/23 (17%)
Injected + mock ethanol treatment	19/30	2/19 (11%)
Injected + 5 µm 4-OHT treatment	22/60	10/22 (45%)
Uninjected	24/30	0/24 (0%)

In summary, in this study we applied our CRISPR/Cas9 short homology directed targeted integration strategy^25^ to generate a set of zebrafish proneural Cre drivers that express functional Cre recombinase in the pattern of the endogenous targeted *ascl1b*, *olig2*, and *neurod1* genes. Somatic targeting of 2A-CreERT2 into *neurod1* in ubi:Switch embryos showed switching in the nervous system after treatment with tamoxifen, suggesting our approach can be used to generate endogenous CreERT2 lines that express inducible Cre recombinase activity. We were able to efficiently recover precise in-frame 2A-Cre recombinase germline integration alleles after screening a minimum of only 20 adult F0 fish. The frequencies of allele recovery were similar to our previous report describing efficient knock in of 2A-RFP and 2A-EGFP reporter cassettes at eight zebrafish loci^25^. Although *ascl1b*, *olig2*, and *neurod1* do not show haploinsufficiency, for other loci it may be beneficial to maintain the wild type endogenous gene expression in the targeted allele. Previous work showed homozygous *otx2:CreERT2* embryos generated by CreERT2 integration upstream of the zebrafish *otx2* initiating ATG were morphologically normal, but endogenous *otx2* expression was reduced^24^. In contrast to our method, the frequency of on target integration using 1000 bp of 5′ homology was 3%, and the targeted cassette did not contain a secondary marker for rapid visual genotyping. Nagy et al. (2019) inserted Cre before the translation termination codon in the mouse megakaryocyte/platelet-specific *Gp1ba* gene, which led to a decrease in GPIbα protein expression and multiple platelet defects^48^. These studies suggest the potential for targeted integration to negatively impact endogenous gene expression will be specific to gene and location, and may require testing alternative design strategies. Our efficient and simplified method for generating zebrafish transgenic Cre driver lines by CRISPR targeted integration can be readily applied to other loci, expanding opportunities to develop new recombinase genetic tools for investigation of specific cell types, developmental stages, or disease states.

## Methods

### Ethics declarations and approval for animal experiments

The zebrafish research in this study was performed according to the Guidelines for Ethical Conduct in the Care and Use of Animals^49^. All zebrafish experiments were carried out in accordance with Iowa State University Animal Care and Use Committee IACUC-18-279 and IACUC-20-058 approved protocols. All methods involving zebrafish were in compliance with ARRIVE guidelines^50^, and the American Veterinary Medical Association (2020) and NIH guidelines for the humane use of animals in research.

### Zebrafish strains and maintenance

Zebrafish (*Danio rerio*) were maintained on an Aquatic Habitats (Pentair) or Aquaneering aquaculture system at 27 °C on a 14 h light/10 h dark cycle. The wild type strain WIK was used to generate the knock in lines and was obtained from the Zebrafish International Resource Center (https://zebrafish.org/home/guide.php). The *casper* mutant fish and ubiquitously expressed floxed EGFP-mCherry line ubi:Switch (*Tg(ubi:loxP-EGFP-loxP-mCherry)*) (Mosimann et al. 2011) were obtained from the lab of Dr. Leonard Zon (Harvard). *casper*; *ubi:switch* and *nacre; ubi:switch* fish were established by crossing *ubi:switch* into *casper*, followed by additional backcrosses to *casper*.

### pPRISM-2A-Cre and -CreERT2 vectors and targeted integration

The pPRISM-2A-Cre vector was designed to be compatible with our previously described short homology-based CRISPR/Cas9 knock in strategy^25^. The cassette in the pPRISM-2A-Cre vector has two functional units: (1) a 2A-Cre cDNA cassette and (2) a secondary fluorescent reporter. The 2A-Cre unit is composed of a self-cleaving 2A peptide from porcine teschvirus-1, the Cre recombinase cDNA, and an ocean pout transcriptional termination and polyadenylation sequence (*Zoarces americanus*, Gibbs and Schmale, 2000). The viral 2A peptide has been used extensively in zebrafish for production of multicistronic messenger RNAs^51^, allowing release of Cre protein from the short endogenous gene’s polypeptide. The secondary reporter cassette for visually track transgenic embryos contains the gamma crystallin (*γ*-*cry*) promoter, mini-intron, nls-EGFP, bovine growth hormone transcriptional termination and two SV40 polyadenylation sequences (Yang and Cvekl 2005; Clark et al*.* 2011; Kim et al*.* 2011). Flanking the entire sequence are universal sgRNA (UgRNA) sites for Cas9 induced double strand breaks to liberate the short homology arms and drive integration of the Cre-reporter targeting cassette^25^. The pENTR_D_CreERT2 vector was a gift from Dr. Christian Mosimann (University of Colorado Anschutz Medical Campus). Using a combination of restriction enzyme digestion, PCR, and New England Biolab Hi-Fi cloning, the Cre cDNA in the pPRISM-2A-Cre vector was removed and replaced with CreERT2.

CRISPR sgRNAs target sites in the coding sequence were selected and tested for efficient indel formation by co-injection of 25 pg sgRNA plus 300 pg Cas9 mRNA into 1 cell stage wild type WIK zebrafish embryos, followed by PCR amplification of the targeted exon and analysis of heteroduplex formation by gel electrophoresis. 48 bp 5′ and 3′ homology arms were designed and cloned into the pPRISM-2A-Cre vector as described^25^. sgRNA and homology arm oligonucleotide sequences are listed in Supplementary Table S1. For targeted integration the injection mix contained 25 pg of genomic sgRNA, 25 pg of UgRNA, 10 pg of targeting vector, and 300 pg Cas9 mRNA. Wild type WIK zebrafish embryos at the 1-cell stage were injected with 2 nl of the injection mix and screened at 3 dpf for fluorescent secondary marker expression. All injected embryos showing EGFP expression in the lens were selected and raised to adulthood.

### *neurod1*-2A-CreERT2 somatic targeting and tamoxifen treatment

The *neurod1* 48 bp 5′ and 3′ homology arms were cloned into the pPRISM-2A-CreERT2 vector. *nacre; ubi:Switch* embryos were injected with 25 pg of genomic sgRNA, 25 pg of UgRNA, 10 pg of targeting vector, and 300 pg Cas9 mRNA. At 6 hpf the embryos were placed in 5 um 4-hydroxytamoxifen (Sigma H6278) in ethanol or ethanol alone and placed in a light-blocking container at 28 °C. The tamoxifen solution was replaced on day two and day three with fresh solution.

### Isolation of stable integration alleles and genome/vector junction analysis

To identify adult F0 founders, adult fish were outcrossed to wild type WIK and at least 75 embryos were screened for lens-specific EGFP expression. EGFP positive F1 embryos were selected for genome/vector junction analysis. Genomic DNA was extracted by digestion of single embryos in 50 mM NaOH at 95 °C for 30 min and neutralized by addition of 1/10th volume 1 M Tris–HCl pH 8.0. Both 5′ and 3′ junctions were amplified by PCR using the primers listed in Supplementary Table S2, TOPO-TA cloned and sequenced. EGFP positive F1 embryos from transmitting founders were raised to adulthood and confirmed by fin clip. F1 adult animals with precise integration events were outcrossed to wild type WIK and single F2 adults were used to establish independent transgenic lines.

### In situ hybridization and live embryo imaging

Embryos were obtained from *ascl1b-2A-Cre/*+, *olig2-2A-Cre*+ and *neurod1-2A-Cre/*+ outcrossed to wild type WIK, and placed in embryo media with 0.003% 1-phenyl 2-thiourea (PTU) at 24 h post fertilization to block pigment production. Whole mount in situ hybridization was performed as described previously^52^. 3 dpf larvae were fixed overnight at 4 °C in 4% paraformaldehyde or 4% paraformaldehyde/4% sucrose in PBS. *neurod1*, *olig2* and *ascl1b* cDNAs were cloned by RT-PCR using total RNA isolated from 3 dpf embryos and SuperScript III (Invitrogen). Primers for reverse transcription and PCR are listed in Supplementary Table S2. Digoxigenin-labeled probes were generated from linear plasmid DNA using DIG RNA Labeling Mix (Roche) and hybridized probes were detected with anti-digoxigenin antibody (Anti-Digoxigenin-AP, Roche) and NBT/BCIP (Roche). Larvae were imaged on a Zeiss Axioskop 2 and photographed with a Cannon Rebel T3 camera. Larvae for live imaging were placed in embryo media with 0.003% 1-phenyl 2-thiourea (PTU) at 24 h post fertilization to block pigment production. At 2 or 3 dpf the PTU treated embryos were anesthetized in 160ug/ml tricaine methane sulfonate and mounted in 1.2% low-melting agarose/160ug/ml tricaine methane sulfonate. Images were captured on a Zeiss LSM 700 or Zeiss LSM 800 laser scanning confocal microscope.

## Supplementary Information

Supplementary Figures.

Supplementary Tables.

## Data Availability

All DNA constructs and transgenic zebrafish lines reported in this study are available on request.
