# Supplementary material for: Endogenous zebrafish proneural Cre drivers generated by CRISPR/Cas9 short homology directed targeted integration
Source: Sci Rep. 2021 Jan 18;11:1732. doi: 10.1038/s41598-021-81239-y (PMC7813866; doi:10.1038/s41598-021-81239-y)
Supplement: Supplementary file 1 — Supplementary Figures. [file 41598_2021_81239_MOESM1_ESM.pdf]

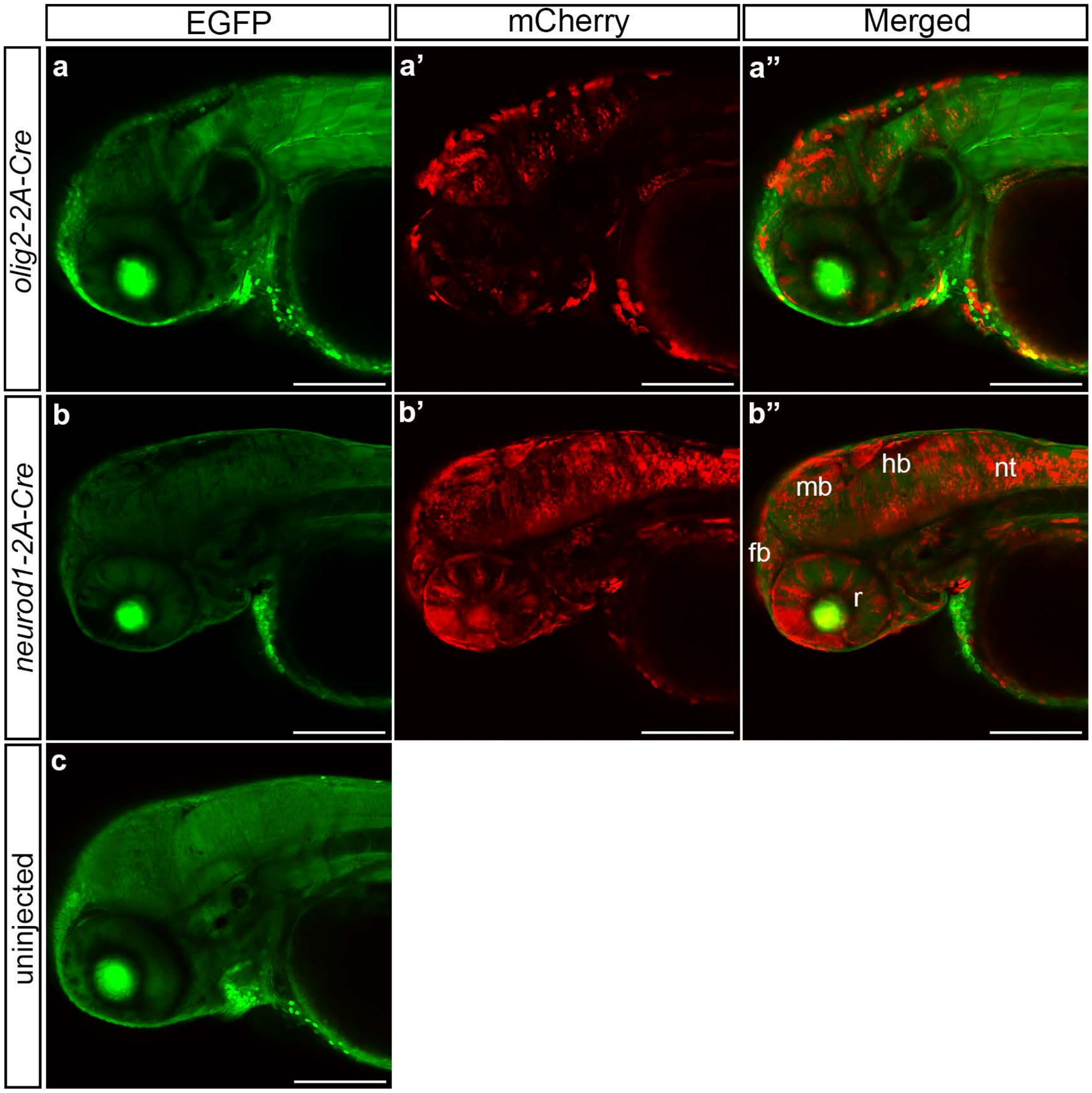

Supplementary Figure S1. CRISPR/Cas9 targeted integration of *olig2-2A-Cre* and *neurod1-2A-Cre* cassettes leads to efficient Cre-*loxP* recombination in F0 mosaic animals. (a) 3 dpf *ubi:Switch* embryos that were injected with Cas9 mRNA, *olig2* exon 2 sgRNA, the universal guide RNA UgRNA, and *olig2-2A-Cre* donor vector show a switch from EGFP to mCherry expression in the midbrain, hindbrain, and neural tube, indicating Cre-mediated excision of the floxed EGFP cassette. (b) Embryos injected with Cas9 mRNA, *neurod1* exon 2 sgRNA, the universal guide RNA UgRNA, and the *neurod1-2A-Cre* targeting vector show the EGFP to mCherry switch in presumed neurons throughout the entire developing central nervous system, including the retina. (c) Uninjected animals express only EGFP. *EGFP,* green fluorescent protein; *fb,* forebrain; *hb*, hindbrain; *mb,* midbrain; *nt*, neural tube; *r,* retina. Scale bar 100 μm.

5’ junctions

```
           ascl1b          48 bp homology arm             vector
Reference   TACCGAACGGAGCGGCGAACAAGAAGATGAGCAAAGTGGAGACGCTGCGCTCCGGGCTTCCC
F0#3, F1#1  TACCGAACGGAGCGGCGAACAAGAAGATGAGCAAAGTGGAGACGCTGCGCTCCGGGCTTCCC

            olig2          48 bp homology arm             vector
Reference    ACTCCTCAAAGCCCCGTCAGCCGGCGCTGGGCCTCTGGGCGCGGGTTTCCAGCAGCTTCCC
F0#20, F1#1  ACTCCTCAAAGCCCCGTCAGCCGGCGCTGGGCCTCTGGGCGCGGGTTTCCAGCAGCTTCCC
F0#20, F1#2  ACTCCTCAAAGCCCCGTCAGCCGGCGCTGGGCCTCTGGGCGCGGGTTTCCAGCAGCTTCCC

           neurod1         48 bp homology arm             vector
Reference   AAACAGCAAGTGCTTCCTTTTCCGCTCTTCCCTACTCCTACCAGACGCCCGGTCTGCTTCCC
F0#1, F1#1  AAACAGCAAGTGCTTCCTTTTCCGCTCTTCCCTACTCCTACCAGACGCCCGGTCTGCTTCCC
F0#1, F1#2  AAACAGCAAGTGCTTCCTTTTCCGCTCTTCCCTACTCCTACCAGACGCCCGGTCTGCTTCCC
```

3’ junctions

```
            vector          48 bp homology arm           ascl1b
Reference   AGGAAGCCGTGGAGTACATCCGAGCCCTGCAGCAGCTGCTGGACGAGCACGACGCCGTGT
F0#3, F1#1  AGGAAGCCGTGGAGTACATCCGAGCCCTGCAGCAGCTGCTGGACGAGCACGACGCCGTGT

             vector         48 bp homology arm            olig2
Reference    AGGAAGCTGGGGTGCGGGGATCCCTTGTCCGTGTAGCATGTGCCAGGTGCCTCCGTCTCA
F0#20, F1#1  AGGAAGCTGGGGTGCGGGGATCCCTTGTCCGTGTAGCATGTGCCAGGTGCCTCCGTCTCA
F0#20, F1#2  AGGAAGCTGGGGTGCGGGGATCCCTTGTCCGTGTAGCATGTGCCAGGTGCCTCCGTCTCA

            vector          48 bp homology arm          neurod1
Reference   AGGAAGTTCCCAGCCCTCCGTACGGTACAATGGACAGCTCTCACATCTTTCACGTCAAGC
F0#1, F1#1  AGGAAGTTCCCAGCCCTCCGTACGGTACAATGGACAGCTCTCACATCTTTCACGTCAAGC
F0#1, F1#2  AGGAAGTTCCCAGCCCTCCGTACGGTACAATGGACAGCTCTCACATCTTTCACGTCAAGC
```

Supplementary Figure S2. Sequence analysis of genome/vector junctions in F1 *ascl1b-2A-Cre*, *olig2-2A-Cre* and *neurod1-2a-Cre* transgenic F1 adults. 5’ and 3’ genome/vector junctions were PCR amplified, TA cloned and sequenced. Sequences were aligned to the reference sequence expected following a precise integration event. Red nucleotides represent the 48 bp homology arms used in the targeting vector. Blue letters represent additional nucleotides used to maintain the reading frame between the targeted exon and 2A-Cre cassette.

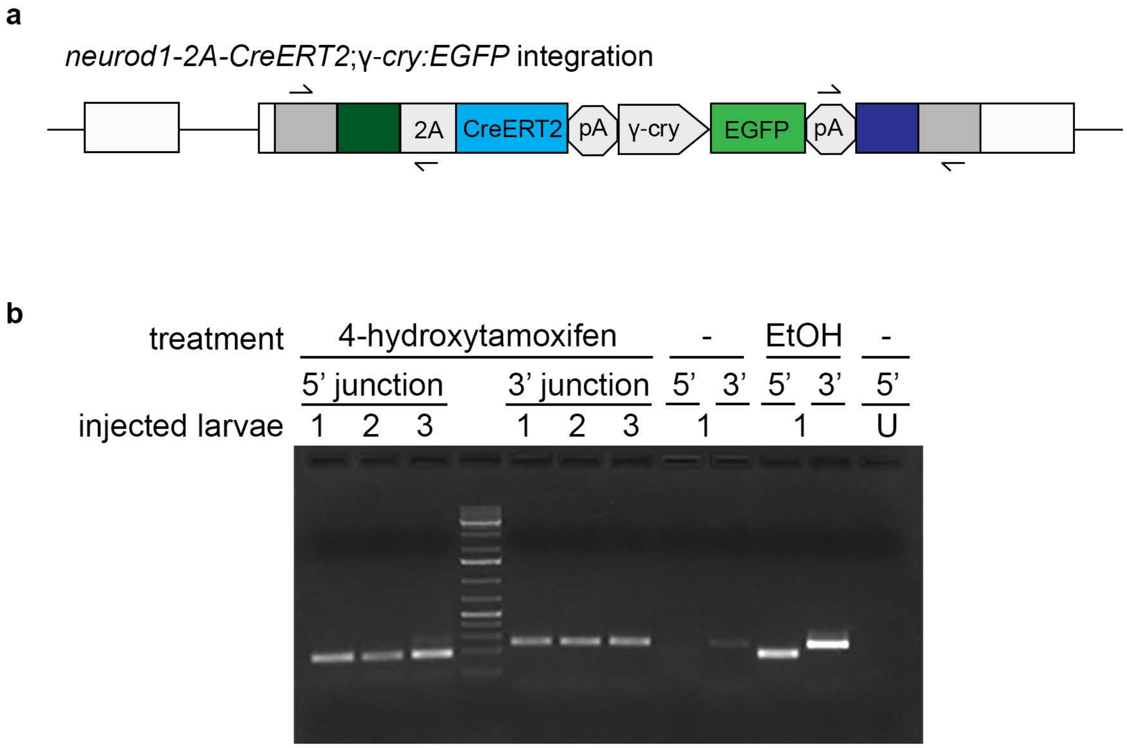

Supplementary Figure S3. ***neurod1-2A-CreERT2*-genomic DNA junction analysis in *nacre ; ubi:Switch* 3 dpf larvae after somatic targeting.** Genomic DNA from larvae injected with CRISRP/-Cas reagents to integrate the 2A-CreERT2 cassette into *neurod1* exon two and treated with tamoxifen, mock treated, or not treated were analyzed for 5’ and 3’ junctions by PCR. (a) Diagram illustrating integration at the *neurod1* genomic target site and location of primers for 5’ and 3’ junction analysis. (b) Both 5’ and 3’ junction PCR products were amplified from genomic DNA from all larvae injected as embryos. Gel shows results from 3 larvae treated (4-hydroxytamoxifen), 1 larvae mock treated (EtOH), and 1 larvae no treatment (-). U, PCR on uninjected control larvae did not result in a 5’ amplification product.
